# Cyber-Security Threats and Side-Channel Attacks for Digital Agriculture

**DOI:** 10.3390/s22093520

**Published:** 2022-05-05

**Authors:** Adel N. Alahmadi, Saeed Ur Rehman, Husain S. Alhazmi, David G. Glynn, Hatoon Shoaib, Patrick Solé

**Affiliations:** 1 Department of Mathematics, Faculty of Science, King Abdulaziz University, Jeddah 21589, Saudi Arabia; hsalhazmi@kau.edu.sa (H.S.A.); hashoaib@kau.edu.sa (H.S.); 2 College of Science and Engineering, Flinders University, Adelaide, SA 5001, Australia; david.glynn@flinders.edu.au; 3 I2M (Centrale Marseille, CNRS, Aix-Marseille University), 13009 Marseilles, France; sole@enst.fr

**Keywords:** side-channel attacks, vulnerability analysis, power analysis attack, security threats, cryptography, digital agriculture, smart agriculture, smart farming

## Abstract

The invention of smart low-power devices and ubiquitous Internet connectivity have facilitated the shift of many labour-intensive jobs into the digital domain. The shortage of skilled workforce and the growing food demand have led the agriculture sector to adapt to the digital transformation. Smart sensors and systems are used to monitor crops, plants, the environment, water, soil moisture, and diseases. The transformation to digital agriculture would improve the quality and quantity of food for the ever-increasing human population. This paper discusses the security threats and vulnerabilities to digital agriculture, which are overlooked in other published articles. It also provides a comprehensive review of the side-channel attacks (SCA) specific to digital agriculture, which have not been explored previously. The paper also discusses the open research challenges and future directions.

## 1. Introduction

The human population has increased exponentially in the last century. It is estimated that it will peak at 10.9 billion by 2100 [1]. The quality and quantity of global food resources have improved mainly due to technological innovations in genetic engineering in the last fifty years. Genetic engineering helps develop seeds and plants that can grow with less water and produce more nutrients to meet the demands of a growing population. Digital agriculture is the next technological innovation for the sustainable production of food in the agriculture sector [2]. Countries are combating desertification, for example the Saudi Green Initiative (an extension of Saudi Vision 2030), where four million lemon trees that rely on recycled water for irrigation are being planted, as well as hundreds of millions of other trees that should modify the climate and aid farming.

Digital agriculture is not immune to cyber-attacks, which can range from controlling a heating and ventilation system of a vertical farm to controlling a drone used for spraying crops. In recent times, cyber-attacks on the Florida water system [3], Lion (an Australian beverage company with business in diary and drinks), wool broker software [4], and JBS [5] (the world’s largest meatpacker) have made headlines around the world. This has highlighted the vulnerabilities in digital agriculture and potential disastrous effects on the general population in terms of supply, labour, and cost.

Typically, malicious actors target cheaper and more accessible pathways that could be vulnerable, involving humans, devices, software, processes, or technologies, under-protected by the user, but having very serious implications. The authors in [6] audited six dairy farms in Finland, and it was found that most of the networking equipment was physically not secured and default credentials were used, which could be easily compromised. The threat actors have also evolved from amateurs to sovereign states with virtually unlimited resources. The 2022 World Economic Forum survey put cyber-security failure in the top 10 risks, worsening in the COVID-19 crisis, while at the regional level, it is in the top 5 risks [7].

Cyber-security is becoming common vernacular due to the plethora of attacks on digital infrastructure. Nakhodchi et al. [8] performed a bibliometric analysis of publications in the security and privacy of smart farming and found 141 articles related to agricultural cyber-security. Recently, some survey papers have discussed the security threats and vulnerability assessment for digital agriculture [9,10,11,12,13]. Most research revolves around traditional threats and mitigation, in particular hardware and software security and cryptography.

Typically, in an information network, the confidentiality of data is achieved through encryption, which scrambles the plain text into unreadable (cipher) text. Encryption is physically implemented in electronics. Power consumption, electromagnetic emissions, timing, and thermal signatures provide useful information that may reveal the encryption standard and keys to break the encryption. This extraction of information from the operation of physical hardware is termed side-channel attacks (SCAs) [14].

Recently, researchers have turned their attention to side-channel attacks (SCAs) on traditional computer networks, primarily investigating cryptographic information leakage. To the best of the authors’ knowledge, there is no paper dedicated to side-channel attacks on digital agriculture or smart farming. The closest work is about SCAs on the Internet of Things (IoT) [15]. This research article would be the first to discuss side-channel threats, attacks, and their implications for digital agriculture. We aim to initiate a conversation in this relatively unexplored direction.

This paper has the following contributions:

- We critically evaluated the existing literature on the cyber threats to digital agriculture.
- Details of SCA threats to digital agriculture and their implications are presented.
- We discuss the cyber-threats and related open challenges, both technical and non-technical, concerning digital agriculture.

The remainder of the paper is organised as follows: Section 2 defines digital agriculture and its different applications. Section 3 details threats to digital agriculture. Section 4 gives an overview of side-channel attacks, their different variants, and threats with examples in digital agriculture. Section 5 discusses the research challenges, and Section 6 presents the conclusions.

## 2. Digital Agriculture

Agriculture is the lifeline of humans and provides not only food, but also generates employment. The high demand and sustainable food production, shortage of skills, and efficient use of limited environmental resources demand the modernisation of the centuries-old agricultural sector. Digital agriculture (DigAg) (also called smart agriculture/farming) is the use of various digital devices to monitor, assess, and manage environmental parameters that could affect food production (crops, fruit, etc.) [2]. The environmental parameters could be soil condition, water use, moisture content, plant and crop diseases, weather conditions, pests, pollination, nutrition, and the irrigation system. Digital devices such as smartphones, various sensors, global position systems (GPSs), robotics, and drones could be utilised to extract valuable data and analyse and make effective decisions to increase food production with less human resources and intervention.

**Figure 1:**
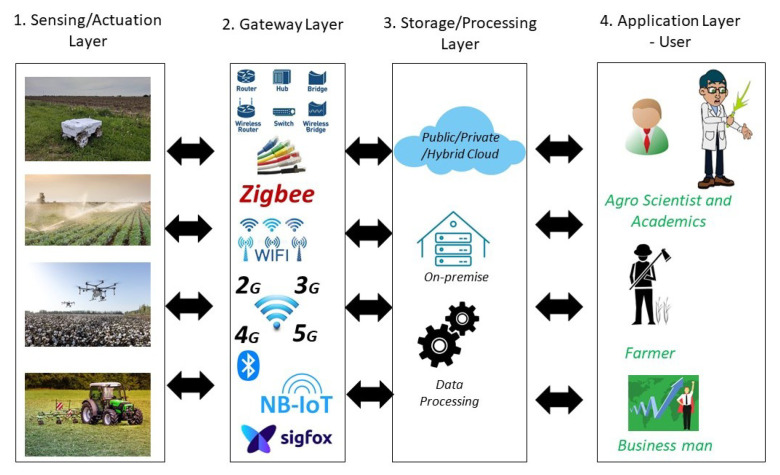
An overview of digital agriculture and its various applications.

Figure 1 shows an overview of digital agriculture and its various components. Broadly, it can be split into four separate layers.

1. Layer 1 is a sensing layer with different sensors to monitor the plants or environmental factors ranging from soil to weather conditions. Sensors would vary for different applications and use cases. These sensors are typically inexpensive, have small computation and battery power, are deployed in the field, and are primarily unattended in a hostile environment. The same layer can have actuator functionalities to perform a specific operation, such as water control or spraying via drones.
2. Layer 2 is the gateway layer, where gateways provide an interface between the Internet and sensors. Typically, wireless communication is used to connect sensors. Depending on the application requirements, Zigbee, WiFi, Bluetooth, NB-IoT, Sigfox, LoRa, 5G, or satellite communication are used. The forwarding devices such as switches/access points are part of this layer.
3. Layer 3 is the storage or processing layer. An in-house data storage or cloud solution could be used.
4. Layer 4 is the application layer, where all the users see or control the sensors. Useful analytics are extracted from the data, and based on this, an informed action is performed. The end-user could be a farmer, an agroscientist, a broker, a trader, a government official, or a business.

The standard IoT model combines Layers 3 and 4 into one layer and calls it the application layer. For digital agriculture, it should be split into two, as multiple users can use the same data for their individual purposes. Further splitting it into two layers makes the threat analysis easier and more accountable for data usage or malicious use.

DigAg (pronounced “Didge-Ag”) has several applications. Some are crop management, automation, precision agriculture [16], and monitoring activities. The latter include watching over or controlling irrigation and water quality [17], soil [18], weather, farm, pests, and diseases [19]. The subsequent sections highlight the use of DigAg in smart irrigation [20] and intelligent machinery [21], discussing some of the threats that malicious actors could exploit.

### 2.1. Application—Smart Irrigation System

Water is, of course, essential for life, especially so in the desert. Global warming, growth of the population, and inefficient use or scarcity of water demand smart irrigation systems. Various kinds of sensors (temperature, moisture, ultrasonic, etc.) can be used to monitor the water level, soil moisture, plant/crop condition, and weather to optimise the use of precious water. These sensors are deployed remotely, battery-powered, and have low computational power. An actuator is deployed based on the sensory data. Aerial systems are also used to monitor soil and moisture content using cameras (thermal or RGB) deployed on drones or low-Earth-orbit satellites. This creates a wide attack surface that is difficult to defend against and is vulnerable to exploitation. The threats to smart irrigation and sensors can range from physical compromise to falsifying the data. As mentioned in Table 1, the traditional threats are equally applicable to different layers of a smart irrigation system.

### 2.2. Application—Intelligent Machinery in Agriculture

An intelligent agricultural machine can use sensors and computer logic to control and operate the equipment to achieve a defined goal on the ground with minimum human intervention. A large agricultural paddock can be divided into small plots for cultivation. The soil, moisture, precise seed planting, and land level variances make it difficult to achieve maximum productivity with limited manual or semi-autonomous resources. For example, analysing the soil and moisture contents in real-time and precisely applying fertiliser or other chemicals based on need are time-consuming in a manual operation and are dependent on the skilled farmer. An intelligent machine fills the skill gap and works virtually 24/7. It could be used in all aspects of agricultural tasks such as seed planting on waterways, harvesting, applying fertilisers, monitoring the health of crops, and levelling and ploughing the fields.

A fully automated system should have the intelligence to know its precise location, find the path, be equipped with a safety system, and activate monitoring, analysis, and actuation related to cultivation. The intelligence can be achieved by integrating different sensors, actuators, and communication systems. The attack surface spans multiple systems, and exploiting a vulnerability in any part of the machinery could have devastating effects. For example, substandard soil analysis could result in faulty application of chemicals/fertiliser, which will have long-term effects on the productivity of the agricultural field. In some cases, it might not be noticeable even after many weeks, which would make the rectification difficult both in terms of time and money.

## 3. Threats to Digital Agriculture

Various technologies are integrated into one product to perform specific agricultural tasks, as stated in Section 2. For example, an irrigation system has smart sensors/actuators, communication protocols, software, traditional networking devices, and human interaction. These complex systems are often outsourced from diverse vendors produced for many kinds of environment and application, which increases the attack surface, and cyber-criminals can exploit vulnerabilities to compromise one or other parts of the agricultural application. Some of the threats are similar to those in traditional computer or IoT networks, whereas some threats are specific to digital agriculture. Table 1 details the traditional software, hardware, and communication threats that are well investigated in the literature. The mitigation of those threats can be applied to digital agriculture. The following subsections discuss threats that are not explicitly researched for DigAg.

### 3.1. Research and Intellectual Property

In agriculture, years of collaboration and research work among academics, researchers, students, industry partners, funding organisations, and government produce novel solutions to improve the yield and quality of crops in many kinds of environments. Malicious users and state actors are highly interested in this research and IP, which contribute to the national economy and people’s livelihood. Threats to IP can come from an insider, social engineering, technological vulnerabilities/misconfiguration, and data leakage.

### 3.2. Personally Identifiable Information

DigAg systems are a significant investment and are often deployed for long period. Many users access them over their lifetime, such as technicians, farmers, tradespeople, service providers, etc. The personally identifiable information (PII) of these users can be compromised when accessing the system and can subsequently be used for identity theft.

### 3.3. Commercially Sensitive Information

Data theft leads to the extraction of commercially sensitive information, risking small- and large-scale trade relations (farmer to a service provider or international trade). Commercially-sensitive information can be classified [30] as:

- Competitors use production efficiency statistics in their trading decisions, putting primary producers at a competitive disadvantage. Further, growth statistics lead to targeted research and IP theft attacks.
- Land valuation data, pricing data (logistics, supply chain, invoices, etc.), trading volume, sale trends, and growth statistics provide an insight to competitors for a better bargaining edge.
- Poorly defended small agriculture businesses and farms can be targeted to steal invoice information and banking details. These poorly secured businesses become weak links that enable unauthorised access to a large-scale network.

### 3.4. Internet of Things, Robotics, and Aerial Systems

The Internet of Things, robotics, drones, and aerial systems are the enablers of digital agriculture. Sensors and agricultural robots are remotely controlled. The compromise of sensors, actuators, and robots can disrupt their normal operation or, in the worst case, be used in agri-terrorism. Heavy tractors or drones can be used to destroy fields, transport illegal goods, conduct a crime, or make physical attacks by crashing into the target. GPS spoofing and wireless communication vulnerabilities can be exploited to conduct destructive attacks.

### 3.5. Big Data and Machine Learning Threats

A tremendous amount of data is generated from sensors and autonomous farming machines. Machine learning and artificial intelligence techniques provide a unique insight that can be used to improve food production and use the limited resources optimally. However, it raises concerns about the privacy and accuracy of data. Data compromise, falsification, or eavesdropping could skew the ML/AI algorithm, revealing the IP or creating data ownership tension between stakeholders [31].

### 3.6. Supply Chain Threats

Currently, supply chain disruption is a buzz word due to the COVID-19-induced higher inflation. A supply chain is defined as “the design, engineering, production and distribution processes of goods and services from suppliers to customers” [32]. The sourcing of hardware, software, and services from different vendors (globally and locally) creates security vulnerabilities, which should be considered in the design and operation of DigAg products and applications. Researchers have proposed IoT- [33] and blockchain-based [32,34] monitoring and tracking solutions about product information in supply chain management. However, the services part of the supply chain is still not explored, whereas human expertise from third-party sources is vulnerable to insider attacks.

## 4. Side-Channel Attacks

A communication system consists of devices and communication channels. Reasonable security is obtained by accessing devices only with secret credentials and encrypting the communication channel. Side-channel attacks are related to extracting information from the data leakage during the communication or while accessing the system. A related concept to the side-channel is the covert channel used to communicate stealthily either to avoid listeners in the middle or exfiltrate information. Side-channel and covert attacks leverage the physical properties of the hardware, software, or transmission medium to extract useful sensitive information from the internal functioning and operation of the targeted device [35].

In 1996, Kocher [36] demonstrated that timing data in the cryptographic implementation could be used to recover the entire secret key. With the proliferation of smart devices, IoT, sensors, and slack cryptographic implementation on the hardware, various side-channel attacks have been discovered to break the encryption and extract sensitive credentials. Side-channel attacks are categorised into physical and functional [37]. The physical categorisation is based on a measurable quantity that is the by-product of the implementation. Examples are power output, electromagnetic emission, clock timing, user interaction, acoustic, optical, thermal, and network inference (wired/wireless). The functional type is based on the internal functional implementation and computing system working that could leak the data. Examples are memory implementation, CPU/GPU architecture, and software/firmware cryptographic implementation/coding.

**Figure 2:**
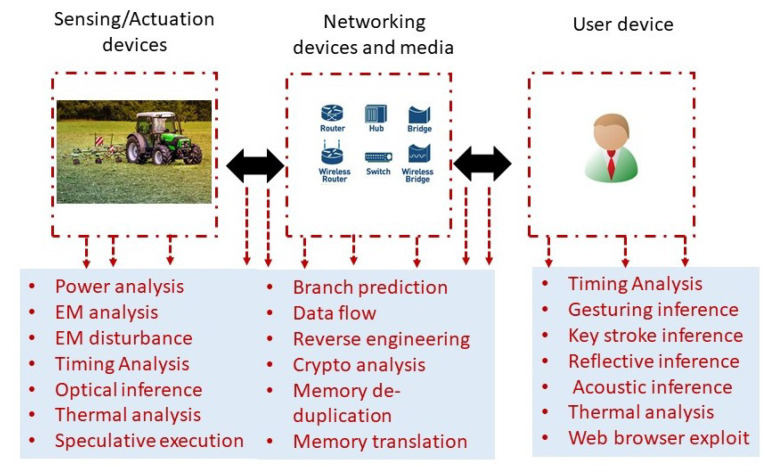
Side-channel attacks for a typical digital agriculture application.

Figure 2 provides a snapshot of various side-channel attacks for a DigAg application. All the physical and functional SCAs are possible on any DigAg applications since most applications are deployed in a harsh environment, not monitored, operated by a non-technical person, and sparsely used. Secret key leakage would lead to all other attacks as mentioned in Section 3.

Table 2 shows the SCAs as reported in the literature. The previously reported SCAs are mostly for computer systems. SCA analysis for IoT devices [15] is closely related to DigAg. The DigAg systems consist of small sensors attached to highly computational devices (drones, autonomous robots). Unlike computer systems, they are unattended and deployed in a harsh environment. Further, their use is infrequent and monitored by a non-technical person. Therefore, the malicious user has limited freedom to play with and change different parameters to reveal sensitive information. A malicious user can install a hardware Trojan to capture and transmit information in the worst-case scenario. For example, power usage SCAs can be easily carried out with physical access to devices. For other applications (e.g., smart homes), the physical access would be relatively difficult compared to digital agriculture, where agriculture equipment is deployed and left in the field.

An SCA is facilitated by physical access. The sensors, actuators, and other agriculture equipment that enable digital agriculture are deployed in the field and occasionally used during the various phases of farming, e.g., land preparation, seed selection and sowing, irrigation, fertilising, and harvesting. The hardware remains in the field or in the shed, which could be easily accessible considering that most farms are out of the city and do not have proper physical security (CCTVs, fencing etc.).

Once a malicious user has physical access, it is at the attacker’s mercy to monitor the side-channels parameter, revealing the cryptographic information or inferring other information, as mentioned in Table 2. For example, a power analysis attack requires power consumption monitoring during a cryptographic operation. A simple power trace of device operations correlated with data-dependent power variations can be used to infer the cryptographic key. A high signal-to-noise ratio (SNR) requires fewer power consumption traces, and close proximity would enable capturing a high SNR trace, making it easy to differentiate traces from one another [15]. In other computing applications, hardware is physically secured, and attackers cannot have prolonged access, unlike in agriculture. Therefore, different variants of SCAs can be easily initiated, as given in Table 2.

Further, low-cost and re-purposed hardware devices (sensors, actuators) do not have a built-in security mechanism to monitor their status, usage, or access to the memory. A secure memory (EEPROMs) is required to store the cryptographic keys securely. Physical unclonable functions (PUFs) could be used for tampering protection and low-cost authentication without relying on secure storage [54]. PUFs can derive secrets from the integrated circuit and be used in low-cost authentication and key generation, minimising secure storage requirements.

## 5. Research Challenges and Future Directions

Most new technology products are developed and commercialised to capture the market quickly. Many devices and sensors are not made explicitly for DigAg applications, but are modified to be used in agriculture, where customisation is mostly directed toward utilisation in a harsh uncontrolled outdoor environment. Less thought is given to the security of the devices. Like other technologies, security is usually considered the last priority rather than embedding security into the design phase. This section discusses some of the open challenges, which are still in the early research phase.

### 5.1. Intrusion Detection and Prevention System

Traditionally, intrusion detection and prevention systems (IDS/IPS) are developed for large data networks. However, the requirements of digital agriculture are different and include low-rate sensor data, sparse observation and attenuation, unattended deployment, and remote control. Therefore, new intrusion detection/prevention algorithms should be developed for digital agriculture. Currently, there is no IDS/IPS dataset available for DigAg applications. Existing datasets are either traditional IoT-smart home datasets [55] or computer networks [56]. The availability of an open-source agriculture-based dataset would fuel the research and development of such algorithms and systems. AI algorithms can be handy in the development of IDS/IPS systems. Further, using AI at edge computing and blockchain would be useful to mitigate some of the existing attacks. Considerable work is needed to deploy edge-based IDS systems for digital agriculture.

### 5.2. DigAg Cyber-Security Framework

The digital agriculture revolution is still at an early stage. Continuous Internet connectivity, inexpensive sensors, remote deployment, non-technical end-users, and new applications and use-cases open up new vulnerabilities and security issues. Frameworks and standards are necessary to guide tradesmen, farmers, and businesses to implement security controls. Typically, a framework development takes considerable time as it involves consultation with stakeholders (business, farmers, different agriculture sectors). The framework guides all the stakeholders on implementing security at different levels for various assets (data, devices, applications, etc.). Currently, there is no security framework developed explicitly for DigAg. The National Institute of Standards and Technology (NIST) Cyber Security Framework (CSF) covers IT and operational security [30]. However, it does not capture control over all the IT assets. A closer look at the NIST framework could be a good starting point for developing a security framework specifically for DigAg.

### 5.3. Privacy-Preserving Schemes

Most of the data in the DigAg are related to field work, which users might overlook. Privacy-preserving schemes for DigAg are an emerging area [57]. New privacy-preserving schemes need to be developed tailored for digital agriculture to protect the data from the malicious user in all aspects such as data privacy, data analytics, data utility, and overall system efficiency. New privacy-preserving schemes would mitigate IP theft, PII, and commercially sensitive information.

### 5.4. Vulnerability and Threat Analysis

DigAg devices and IT requirements are different for various applications. Hardware and software from multiple vendors are integrated into one particular solution, which increases the attack surface. Before integrating the devices, a thorough vulnerability and threat analysis should be performed, including the side-channel attacks, which are difficult to analyse and typically not covered in the cyber-security frameworks. Each hardware system should be analysed in the context of its use and threats, whether physical, hardware, or software-related.

### 5.5. Cyber Awareness and Incidence Response

Cyber attacks are inevitable. It is not a question of if, but when. Previous security breaches have shown that malicious users exploit technical vulnerabilities through an unintentional harmless action by the end-users. Humans are always the weakest link. Cyber awareness and training of end-users, installing security appliances (firewall, antivirus software, etc.), and being physically aware of an anomaly would stop many of the threats mentioned earlier in Section 2. However, end-users’ continuous engagement and training are challenging, and technology should be developed for this purpose.

The end-user, business, and government should be prepared and equipped with incident response and business continuity plans for unknown attacks in the future. Developing simple incident response and business continuity templates for various DigAg applications would be a cost-effective solution. They would motivate end-users to respond appropriately in case of a breach.

## 6. Conclusions

The digitisation of agriculture paves the way for new applications and new use of technology to increase the yield of crops with less utilisation of resources. Most existing technology is modified and networked to provide innovative solutions to the decades-old agriculture problem. This article provided a generic threat analysis of our four-layer DigAg model. Threats such as IP, PII, etc., which are overlooked for DigAg and side-channel attacks, and their implication were discussed in detail. Finally, open research challenges and future directions were presented. The research challenges should be addressed at an early stage during the development and deployment rather than leaving them to the very end. Else, they would take considerable resources to fix.

## Figures and Tables

**Table 1:** Typical threats to digital agriculture and countermeasures.

	Sensing/Actuation	Gateway	Storage	User
Description	Threats are related to hardware, physical access, damage, firmware/hardware modification, or the wrong actuation to destroy crops.	Threats are related to data in transit and involve network devices and communication protocols. Vulnerabilities can be exploited to sniff out and access data, leading to diverse attacks.	Threats are related to data at rest, either in the cloud or on-premises. The compromise of data could lead to IP theft.	The end-user interface is at Layer 4, and the compromise of credentials through social engineering or malware injection could compromise the whole system.
Threats	Physical attacks, device/sensor or firmware alteration [22], side-channel attacks, eavesdropping [23], booting, physical damage, malicious code, forgery, sleep deprivation attacks [24]	Protocol vulnerabilities [25], authentication, MIM, interference, firmware [26], routing, jamming [27], DoS/DDoS, sniffing attacks	SQL injection, data privacy, IP theft, encryption, confidentiality and integrity, cloud malware injection [28], misconfiguration, flooding attacks in the cloud [29]	Social engineering, phishing, access control, service interruption, insider attacks
Countermeasures Periodic assessment of devices including vulnerabilities, auditing, penetration testing Firmware/software update mechanism to patch security vulnerabilities End-to-end encrypted communication including encrypted drives to keep data inaccessible in the case of device theft Two-factor authentication and secure password recovery mechanisms Block unnecessary services and ports on the devices Avoid device tampering with a physically unclonable function Adaption of a zero-trust model assuming a perimeter-less network				

**Table 2:** Side-channel attacks’ classification and implications for digital agriculture.

SCA Threats	Method and Techniques	Explanation	Implication to DigAg
Microarchitectural (MA) [35]	Speculative execution, branch prediction, data flow analysis, reverse engineering	Malicious user compromises the vulnerability in hardware and software optimisation features of the computer system (CPU, GPU) to reveal secret information.	Most of the equipment is deployed remotely. Therefore, reverse engineering and MA techniques could be used to compromise secret keys.
Power usage [14]	Simple power analysis, correlation power analysis, differential power analysis, USB power analysis [38]	Electronic components utilise energy to execute different instructions. The analysis of energy consumption to execute different instructions can be used to extract secret information.	Like MA, voltage and current analysis could be easily carried out with physical access to the devices.
Electromagnetic emission [39]	EM fault induction, EM disturbance, EM correlation analysis	Electromagnetic emission is related to power usage. Frequency and amplitude are additional information revealed in EM.	Both physical and remote attacks are possible with EM emissions’ analysis.
Clock timing [40]	Timing analysis including differential timing, evict and reload, flush and reload, prime, and count	Clock timing is related to MA side-channel attacks, where internal clock timing analysis could be used to time the execution of an instruction or access the memory.	DigAg applications are deployed in a hostile unmonitored environment. Physically compromising the devices would make it easy to recover secret keys using MA, EM, power usage, and clock timing.
Cryptographic operation [41]	Crypto algorithm attacks [42], deep learning attacks [43], template attacks	Cryptographic algorithms are implemented in hardware or software. MA, EM, power usage, or machine learning could reveal public or private keys.	A combination of MA, EM, power usage, or machine learning techniques can be used to extract secret keys used in public and private cryptography.
Memory operations [44]	Memory deduplication [45], memory translation, electromagnetic disturbance	Memory deduplication is a virtualisation technique in which the same contents across the pages are shared between processors.	Recovery of memory traces by physically accessing the devices used in DigAg applications.
User interaction [46]	Gesture inference, keystroke inference, reflective inference,	User interaction with devices could be used to infer secret information. e.g., how keys are pressed or different gestures while using the device.	These threats are related to users and using the devices to access the DigAg applications.
Acoustic [47,48]	Noise inference [49,50], radio wave induction, vibration inference	Audio leakage of keystrokes, voice recording for voice authentication are some examples	Hardware bugs to record the acoustic data and exfiltrate for later analysis
Virtualisation interface [51]	Multi-tenant cross-talk [52], page fault exploit, virtual machine duplication exploit	The same physical resource is shared among different applications, and the attackers could recover memory traces.	These SCA threats are related to applications and data hosted on the cloud and can lead to IP, PII, and commercial data theft.
Network interface [37]	LED interface, light induction	Physically clamping to the network card or eavesdropping on the wireless communication	Identifying communicating parties—from sending and receiving patterns, behavioural profiling to improve fingerprinting for marketing reasons
Thermal Dissipation [53]	Thermal pattern correlation	Measuring thermal dissipation and correlating it to the workload in the hardware during the execution of instructions.	Thermal cameras and heat maps can be used alongside other SCA techniques on DigAg devices

## Data Availability

Not applicable.
